# Assessment and management of chronic insomnia disorder: an algorithm for primary care physicians

**DOI:** 10.1186/s12875-024-02381-w

**Published:** 2024-04-26

**Authors:** Hugh Selsick, Anna Heidbreder, Jason Ellis, Luigi Ferini-Strambi, Diego García-Borreguero, Chrysoula Leontiou, Michael S.B. Mak, David O’Regan, Liborio Parrino

**Affiliations:** 1 Insomnia and Behavioural Sleep Medicine Clinic, University College London Hospitals, London, NW1 2PG UK; 2 Department of Neurology, Johannes Kepler University Linz, Linz, 4020 Austria; 3 Department of Psychology, Northumbria University, Newcastle, NE1 8ST UK; 4 Department of Neurological Sciences, Università Vita-Salute San Raffaele, Milan, 20132 Italy; 5 Sleep Research Institute, Madrid, 28036 Spain; 6 Idorsia Pharmaceuticals Ltd, Allschwil, 4123 Switzerland; 7 Department of Psychiatry, University of Toronto, Toronto, ON Canada; 8 Faculty of Life Sciences and Medicine, King’s College, London, WC2R 2LS UK; 9 Sleep Disorders Centre, Guy’s Hospital, London, SE1 9RT UK; 10 Sleep Medicine Center, University of Parma, Parma, 43126 Italy; 11 Neurology Unit, Parma University Hospital, Parma, 43126 Italy

**Keywords:** Chronic insomnia disorder, Diagnosis and treatment, Algorithm, Primary care

## Abstract

**Background:**

Primary care physicians often lack resources and training to correctly diagnose and manage chronic insomnia disorder. Tools supporting chronic insomnia diagnosis and management could fill this critical gap. A survey was conducted to understand insomnia disorder diagnosis and treatment practices among primary care physicians, and to evaluate a diagnosis and treatment algorithm on its use, to identify ways to optimize it specifically for these providers.

**Methods:**

A panel of experts developed an algorithm for diagnosing and treating chronic insomnia disorder, based on current guidelines and experience in clinical practice. An online survey was conducted with primary care physicians from France, Germany, Italy, Spain, and the United Kingdom, who treat chronic insomnia patients, between January and February 2023. A sub-sample of participants provided open-ended feedback on the algorithm and gave suggestions for improvements.

**Results:**

Overall, 106 primary care physicians completed the survey. Half (52%, 55/106) reported they did not regularly screen for insomnia and half (51%, 54/106) felt they did not have enough time to address patients’ needs in relation to insomnia or trouble sleeping. The majority (87%,92/106) agreed the algorithm would help diagnose chronic insomnia patients and 82% (87/106) agreed the algorithm would help improve their clinical practice in relation to managing chronic insomnia. Suggestions for improvements were making the algorithm easier to read and use.

**Conclusion:**

The algorithm developed for, and tested by, primary care physicians to diagnose and treat chronic insomnia disorder may offer significant benefits to providers and their patients through ensuring standardization of insomnia diagnosis and management.

**Supplementary Information:**

The online version contains supplementary material available at 10.1186/s12875-024-02381-w.

## Introduction

Insomnia is the most prevalent sleep disorder: one-third of adults (30–36%) report at least one nocturnal insomnia symptom, and prevalence of chronic insomnia disorder is estimated between 6 and 10% [1]. Chronic insomnia disorder is defined as difficulty in falling or staying asleep or experiencing early-morning awakening or non-restorative sleep, three times per week for at least three months, with impairment to daily activity [2]. Chronic insomnia disorder has serious impacts on both physical and mental health, including increased risk for cardiovascular outcomes [3], depression [4], anxiety [5], and neurodegenerative diseases such as Alzheimer’s and dementia [6].

Primary care physicians (PCPs) are typically the first point of contact for care for patients with chronic insomnia across Europe [7]. Guidelines state that insomnia disorder should be diagnosed through a thorough clinical evaluation including a sleep history and hygiene, comorbid conditions, psychiatric history, and substance use [8]. However, lack of knowledge and awareness and insufficient training have been documented as barriers to correct diagnosis and treatment of chronic insomnia among PCPs [9]. In addition, despite being the first line treatment recommended by European guidelines [10], there is currently a shortage of trained Cognitive-Behavioral Therapy for insomnia (CBT-I) practitioners [11, 12], which contributes to undertreatment of chronic insomnia and widespread use of pharmacological interventions such as benzodiazepines and Z-drugs [7]. Z-drugs is a term encompassing zopiclone, eszopiclone, zaleplon and zolpidem which are all approved for insomnia [13]. These medications are often used by PCPs beyond the ≤ 4-week recommendation, and their long-term use carries risks for patients in the form of tolerance, dependence, and misuse [14].

In 2020, an expert committee evaluated guidelines from the previous ten years to develop a more holistic treatment algorithm, which encouraged the evaluation of comorbidities and lifestyle symptoms and recommended CBT-I or individual/group therapy as a first line treatment, followed by medication [15]. However, there is still a need to support PCPs to diagnose and treat chronic insomnia in accordance with current guidelines [10, 16]. For example, such supports could include providing additional information on distinguishing between acute and chronic insomnia disorder, or an aid to identify other sleep disorders such as obstructive sleep apnea.

To address this unmet need, a revised algorithm for diagnosing and treating chronic insomnia disorder was developed with an eye towards the specific unmet needs of PCPs. A survey was then conducted with PCPs to test this algorithm with a focus on its guidance and usability, with the specific objectives of understanding current practices among PCPs treating patients with chronic insomnia disorder, eliciting PCPs’ responses to the algorithm, and incorporating their feedback to further optimize it. The results of the algorithm development, survey, and resulting feedback are reported in this manuscript.

## Methods

### Development of the algorithm

**Fig. 1:**
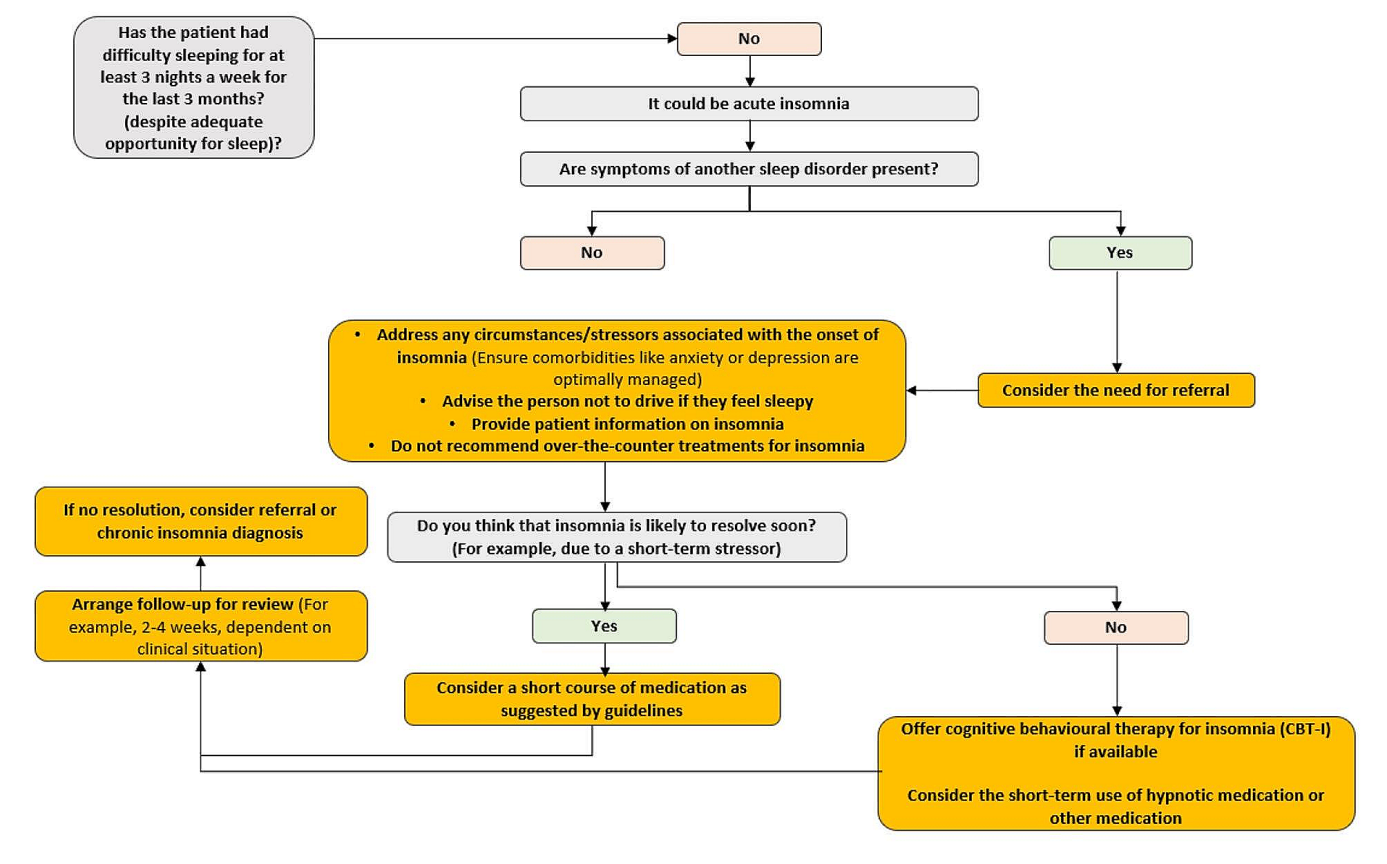
Algorithm tested in research, page 1 – acute insomnia disorder decision flow

**Fig. 2:**
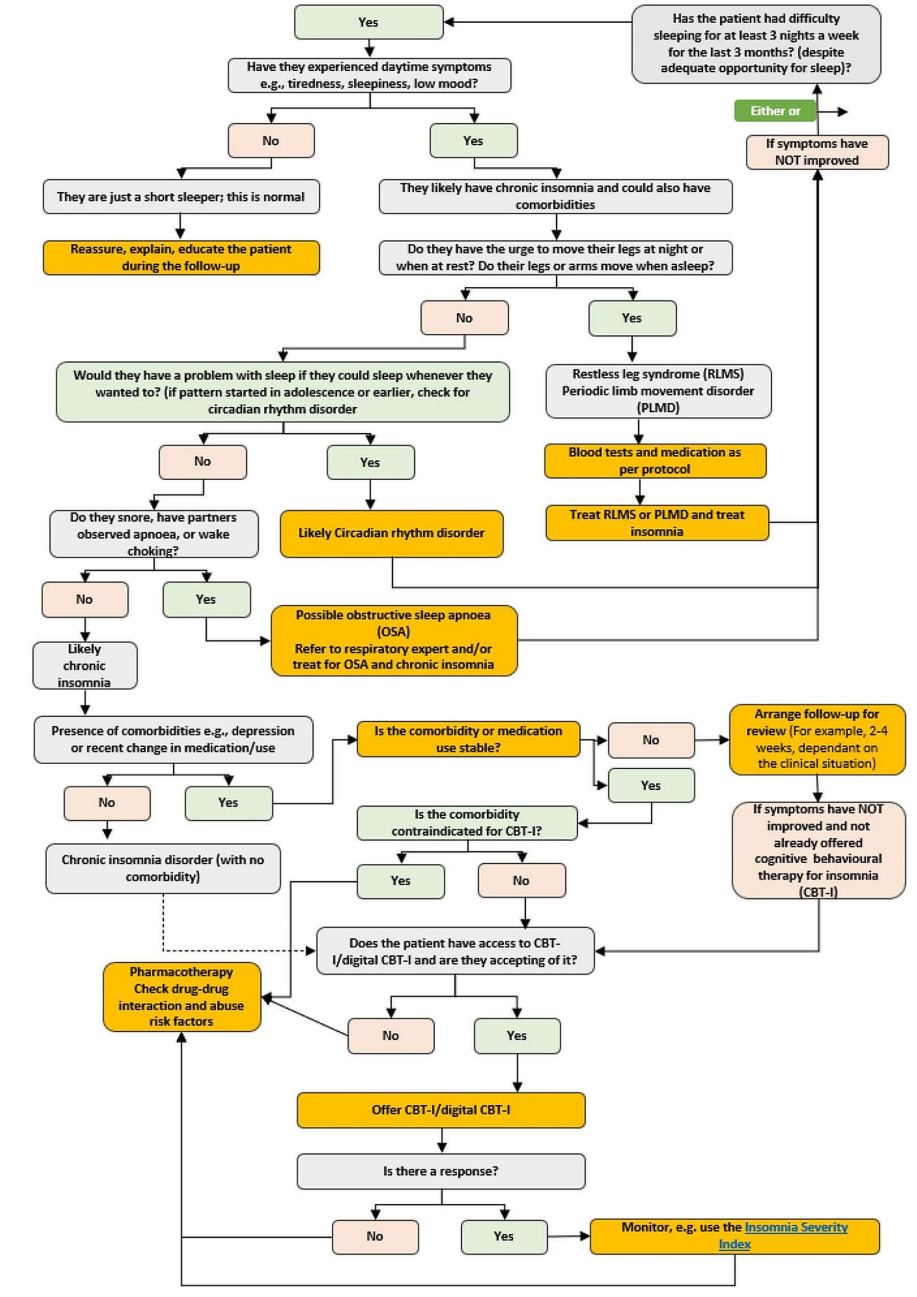
Algorithm tested in research, page 2 – chronic insomnia disorder decision flow

The algorithm tested in this research translates DSM-5 and ICSD-3 definitions of chronic insomnia disorder and the European guidelines [10] into a flow diagram aimed at supporting the diagnosis and management of chronic insomnia. For the purposes of evaluating the tool in the survey, the algorithm was broken into two pages: page 1 showed the acute insomnia disorder decision flow (Fig. 1) and page 2 showed the chronic insomnia disorder decision flow (Fig. 2). The algorithm was developed in a series of workshops by a panel of experts in insomnia and sleep medicine from Europe and North America who drew on existing guidelines, scientific literature, and their clinical experience. The panel clearly defined chronic insomnia disorder to help PCPs differentiate it from acute insomnia, then incorporated steps for the assessment of chronic insomnia (e.g., how to identify comorbid sleep disorders or sleep disorders mimicking insomnia; recommendations for referral to specialists to evaluate comorbid conditions where necessary). For acute insomnia management, guidance from the work of Ellis et al. [17, 18] was taken into consideration, and for chronic insomnia the 2023 update of the European guidelines were applied [9]. The expert panel recommended using the Insomnia Severity Index as a measure of evaluating severity of the disorder, as this is a reliable validated instrument for identifying cases of insomnia and detecting changes in treatment response in clinical patients [19].

### Recruitment and data collection

PCPs were recruited through proprietary vendor panels by Ipsos, using databases of healthcare providers who have indicated a willingness to participate in surveys. Panel providers abided by European Society for Opinion and Marketing Research (ESOMAR) guidelines during recruitment [20]. Initially, they conducted open enrolment and ‘by-invitation-only’ recruitment campaigns, via direct email and through online marketing channels. Prospective panelists were required to provide information on specialty, years in practice, and conditions treated; and subjected to three-factor verification of their identities, qualifications, and areas of specialism.

Participants were pre-qualified via a set of screening questions (Appendix 5) and upon meeting the criteria were given the option to complete the survey after giving written informed consent. Participants were enrolled from 20th January 2023 to 8th February 2023.

### Sample

PCPs from France, Germany, Italy, Spain, and the United Kingdom were invited to participate in the survey. The inclusion criteria were: (1) being active as a PCP for 3–35 years, (2) spending at least 70% of time seeing patients, (3) seeing at least 20 adult chronic insomnia patients in a typical month, and (4) seeing at least 10 patients, per month, on prescription treatment for their chronic insomnia disorder. These criteria were applied to ensure that our sample had experience treating insomnia to comment on the algorithm. 166 PCPs were screened and 60 were excluded for not meeting the inclusion criteria.

### Survey methodology

PCPs completed a 10-minute online survey, which was completed in accordance with the Market Research Society (MRS) Code of Conduct [21], all applicable laws protecting participants’ personal data and responses, and in compliance with ESOMAR [20], European Pharmaceutical Market Research Association [22] and British Healthcare Business Intelligence Association [23] guidelines. Participants were able to unsubscribe from the panel or leave the survey at any time.

Ipsos developed the survey for use in this research in collaboration with Idorsia. Questions included in the survey were written based on the study aims; no validated survey tools were used. First, participants were asked about consultations with insomnia disorder patients, including importance of treating insomnia compared to anxiety and depression, feeling resourced to address patients’ needs, regularity of screening for chronic insomnia, and time to address patients’ needs related to insomnia (see Appendix 5 for full questionnaire). Participants were then shown the algorithm in two parts: an acute insomnia decision flow (Fig. 1) and a chronic insomnia decision flow (Fig. 2) and asked about the algorithm’s utility, including benefits of the tool, ease or difficulty of use, and improvements. Attitudinal questions were answered using either four or five-point Likert scales (e.g., strongly agree, tend to agree, neither agree nor disagree, tend to disagree, strongly disagree) [24]. A sub-sample of participants (*n* = 35) were invited to record an audio open-ended response regarding improvements to the algorithm.

Following completion of the survey, participants were thanked for their participation and provided contact details of the service provider if they had any follow-up questions. Participants were remunerated according to fair market value Incentive rates included the following: 25 EUR for survey completion only and 50 EUR for survey completion and open-end completion in France, 24 EUR and 49 EUR in Germany, 18 EUR and 37 EUR in Italy, 16 EUR and 32 EUR in Spain, and 18 EUR and 36 EUR in the UK, respectively.

### Analysis

Results were analyzed at country level and overall, using SPSS v.29 and Microsoft Excel. Descriptive and directional differences between countries are highlighted but are illustrative in nature due to small country sample sizes. For open-ended feedback provided, a member of the research team sorted responses into overarching themes (e.g., “content” or “visuals”). Themes were then ranked based on frequency of mentions.

## Results

A total of 106 PCPs completed the survey (France *N* = 21, Germany *N* = 22, Italy *N* = 21, Spain *N* = 22, UK *N* = 20) (Table 1). Participants had been qualified for a mean of 15.1 years (SD 9.5) and in a typical month treated a median of 83.0 patients for insomnia disorder (SD 8.2), and prescribed insomnia treatment to a mean of 64.5 patients (SD 5.7).

**Table 1:** Final sample characteristics

	Total (*n* = 106)	Germany (*n* = 22)	France (*n* = 21)	UK (*n* = 20)	Italy (*n* = 21)	Spain (*n* = 22)
	Median (range) Mean (SD)	Median (range) Mean (SD)	Median (range) Mean (SD)	Median (range) Mean (SD)	Median (range) Mean (SD)	Median (range) Mean (SD)
Years of qualification in specialty	12.0 (3.0–35.0) 15.1(9.5)	18.0 (6.0–30.0) 17.8(8.58)	14.0 (3.0–35.0) 18.1(11.7)	9.5 (3.0–25.0) 9.7(4.2)	12.0 (3.0–35.0) 14.9(9.9)	12.0 (3.0–35.0) 14.5(10.5)
Percentage of time spent treating patients	90.0 (61.0–99.0) 90.4(7.62)	95.0 (71.0–99.0) 92.6(6.9)	90.0 (71.0–99.0) 88.0(6.0)	90.0 (71.0–99.0) 91.4(7.3)	95.0 (71.0–99.0) 91.1(8.8)	90.0 (61.0–99.0) 89.1(8.4)
Number patients managed for insomnia in a month	50.0 (20.0-500.0) 83.0(8.2)	40.0 (20.0-500.0) 75.0(21.5)	50.0 (20.0-300.0) 73.3(13.9)	35.0 (20.0-200.0) 55.5(11.8)	75.0 (30.0-500.0) 109.2(23.4)	60.0 (30.0-300.0) 100.3(15.6)
Number patients prescribing treatment for insomnia in a month	40.0 (10–350) 64.5(5.7)	32.5 (10–200) 47.6(9.03)	40.0 (15–200) 58.0(9.7)	30.0 (10–200) 45.0(10.9)	70.0 (23–350) 88.9(18.2)	60.0 (25–250) 81.9(12.4)

### Current practices in diagnosis and management of chronic insomnia

**Fig. 3:**
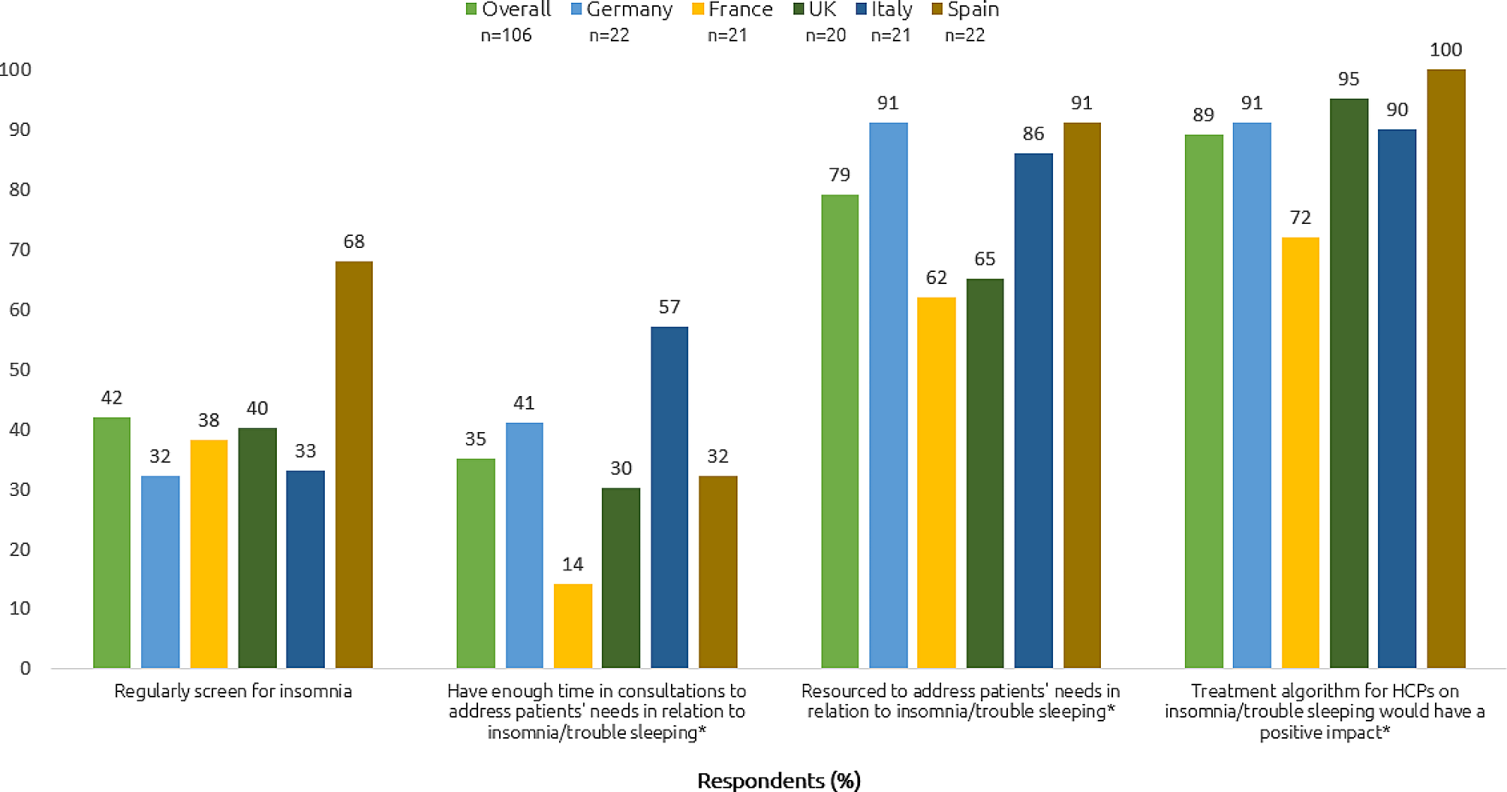
Diagnosis and management of insomnia disorder. Top-2 box scores (tend to agree/strongly agree) are shown

Of the total sample, 52% (55/106) of PCPs reported they do not regularly screen for chronic insomnia disorder. Half (51%, 54/106) tended to disagree or strongly disagree that they have enough time in consultations to address patients’ needs in relation to insomnia/trouble sleeping; this figure was similar across countries except in Italy where only 15% (3/21) tended to disagree or strongly disagree. One in five (21%, 22/106) of participants felt they were not very well or not resourced at all to address patients’ needs in relation to insomnia/trouble sleeping. When asked about the importance of treating anxiety versus insomnia, 19% (20/106) felt insomnia was slightly less important or much less important, similar to 21% (23/106) who felt insomnia was slightly or much more important to treat. When asked about the importance of treating depression versus insomnia, 30% (32/106) felt insomnia was slightly or much less important compared to 10% (10/106) who felt insomnia was slightly or much more important to treat. See Fig. 3 and Appendix 1 for further details.

### Potential impact of the algorithm on PCPs’ practice

**Fig. 4:**
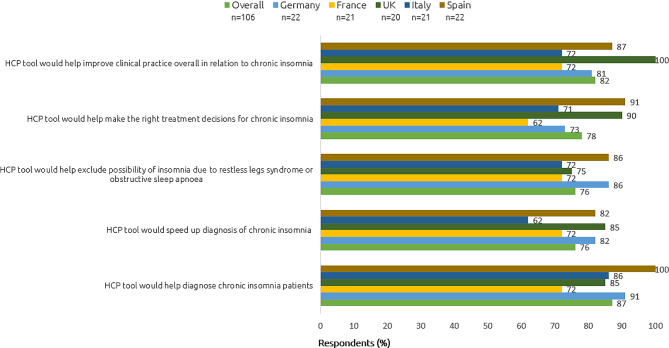
Reactions to algorithm. Top-2 box scores (tend to agree/strongly agree) are shown

Prior to the viewing algorithm, 89% (95/106) of PCPs felt a treatment algorithm would have a very or fairly positive impact on treatment of patients with insomnia/trouble sleeping. After viewing the algorithm, 87% (92/106) of participants tended to agree or strongly agreed it would help diagnose chronic insomnia patients. More than three-quarters (82%, 87/106) tended to agree or strongly agreed that the algorithm would help improve clinical practice overall. The majority tended to agree or strongly agreed that the algorithm would help exclude the possibility of insomnia due to restless legs syndrome or obstructive sleep apnea (76%, 81/106), that it would help make the right treatment decisions for chronic insomnia (78%, 83/106), and that it would speed up diagnosis of chronic insomnia (76%, 81/206). See Fig. 4 and Appendix 2 for further details.

### Utility of algorithm and potential improvements

More than 9 in 10 (92%, 98/106) of PCPs found the first page of the algorithm (acute insomnia) very or fairly useful, and 78% (83/106) found it fairly or very easy to use. The majority (85%, 90/106) found the second page of the algorithm (chronic insomnia) very or fairly useful, and 60% (64/106) found it fairly or very easy to use.

Of those who provided open-ended feedback, suggestions for improvements to page 1 primarily focused on content, suggested by 40% (14/35) of respondents. Improvements to content primarily consisted of making the tool more convenient to use (10/35, 29%), with less frequent mentions regarding simplify text/wording (4/35, 11%), providing more information on treatment (4/35, 11%), and changing the layout (2/35, 6%) 0.23% (8/35) also suggested making improvements for the tool be used in clinical practice or providing information for use with different patient types. Similarly, suggestions for improvements to page 2 were primarily on improvements to content, suggested by 60% (21/35) of respondents. Similar to page 1, feedback was centered around making the algorithm more convenient to use (43%, 15/35) and changing the layout (29%, *n* = 10). Further detail on participants’ answers to questions on usability and improvements to the algorithm is shown in Appendices 3 and 4.

**Fig. 5:**
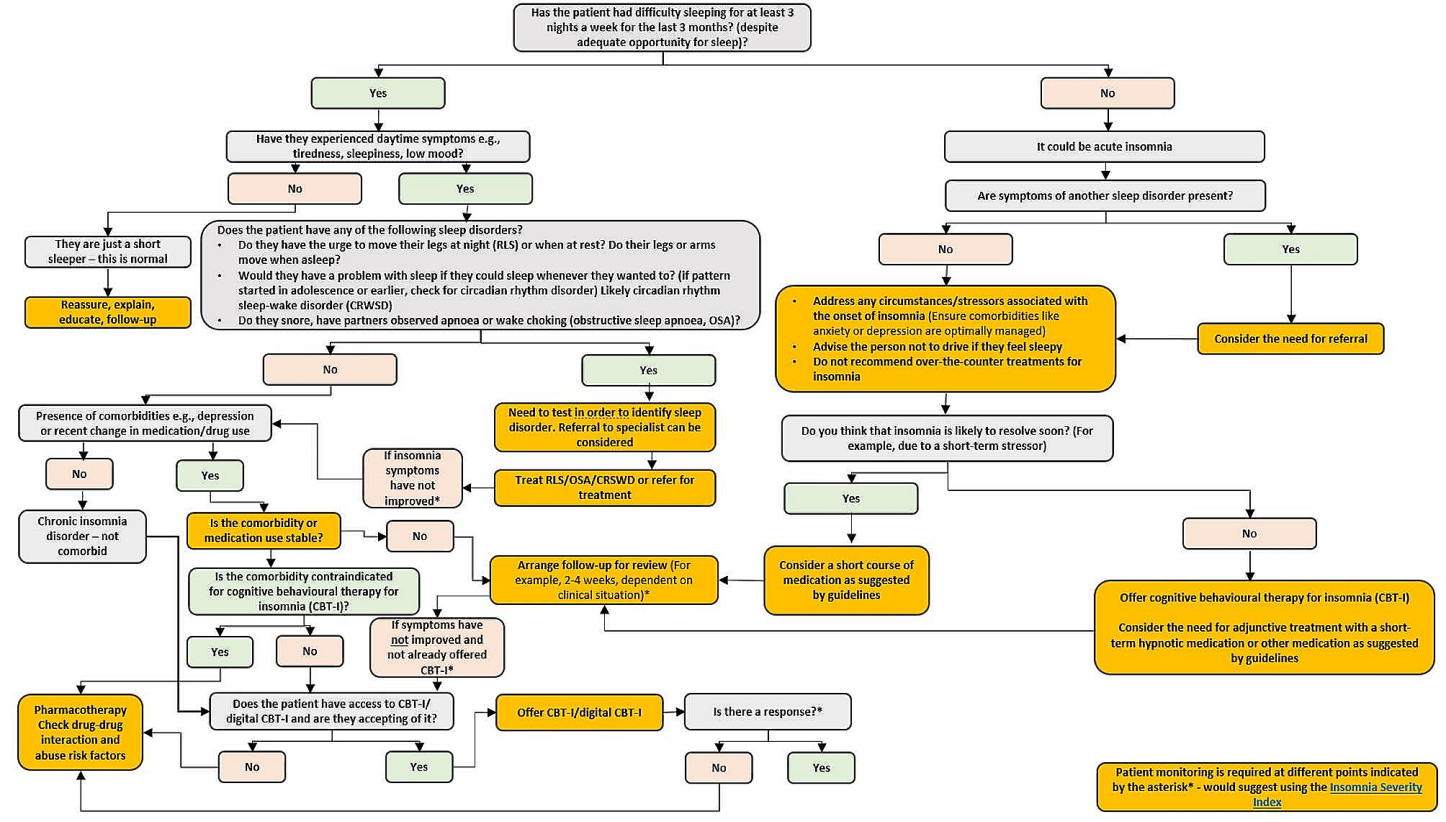
Algorithm - revised

These responses were used to adjust the algorithm (Fig. 5), including putting the tool into one page for easier use, and merging screening for sleep disorders (such as obstructive sleep apnoea or circadian rhythm disorders) into one step, with the resulting advice to the PCP to consider referral to a specialist. In addition, some steps that were considered redundant were removed.

## Discussion

This cross-sectional survey of PCPs with experience treating insomnia aimed to understand their practices and elicit feedback on an algorithm to support insomnia diagnosis and treatment. Our survey confirmed existing literature showing that despite primary care being the first clinical point of contact for insomnia in Europe [7], even among PCPs with experience of treating insomnia, they do not regularly screen for it, as half our sample reported not regularly screening for chronic insomnia. In addition, conditions such as depression may take priority over insomnia, despite being intricately linked – in our sample, 3 of 10 PCPs (32/106) felt treating insomnia was less important, emphasizing the need to educate PCPs that chronic insomnia disorder should be treated independently of other comorbidities.

This survey also confirmed that many PCPs treating insomnia feel they lack time and resources to treat insomnia in their practice, underscoring the need for tools to aid PCPs in the diagnosis and treatment of chronic insomnia. Previous research has noted that in time-poor environments, algorithms are useful in allowing providers to focus on the more demanding details by providing decision support [25]. Our sample was very open to such support: the majority (89%, 95/106) felt a treatment algorithm would have a positive impact on patient management, perceptions of the algorithm were very positive. Most PCPs felt it would speed up their diagnoses and help them to make the right decisions whilst also being consistent with guidelines and diagnostic manuals (e.g., ICSD-3, DSM-5 and ICD-11). This is particularly important for PCPs who may lack specialist knowledge or time available to keep up to date with the latest revisions or local variations in guidance for chronic insomnia diagnosis and management. However, while feedback on the utility of the algorithm was positive, there were clear areas for improvement. Following this feedback, adjustments were made to the proposed algorithm (Fig. 5) which helped to simplify it.

While there appears to be a dearth of research evaluating similar decision support tools for chronic insomnia, there are several studies on such tools in primary care, although they are primarily focused on the impact of electronic clinical decision support tools (eCDST) [26–28]. eCDST allows for providers to enter patient specific information, which is then processed using validated algorithms to make recommendations or issue prompts for the provider to consider [26]. Decision support tools (including but not limited to algorithms) have been shown to have significant effects in screening for common chronic diseases in primary care, but have not been fully validated for acute and uncommon diseases [27]. This algorithm may similarly have the potential to offer PCPs several benefits in their diagnosis and treatment of chronic insomnia, ensuring standardization and streamlined insomnia management in a unified pathway. However, introducing an algorithm alone is unlikely to prompt all the changes required to increase the quality of chronic insomnia diagnosis and treatment in primary care. Increasing PCPs’ awareness of sleep disorders may also help to increase patient access to resources such as sleep centers and insomnia specialists. In addition, evaluation of the algorithm’s use in real-world settings would be required to provide evidence regarding its impact on diagnosis and management of chronic insomnia.

Our findings also highlight the need for the allocation of greater resources to educate on insomnia in primary care. Although there is little existing evidence on the impact of CBT-I training initiatives [29], we suggest that perhaps increasing collaboration between PCP societies and sleep societies to develop training programs on chronic insomnia may be another valuable initiative. In the short-term, online platforms can offer accessible mechanisms for PCPs to request advice on difficult cases, though their effectiveness is yet to be evaluated.

The limitations of this study should be addressed: only cross-sectional data indicating self-reported practices and opinions were presented, which are subject to recall and social desirability bias. Our recruitment criteria may have also influenced the results, for example, it is possible that newly qualified PCPS who were excluded from the research may have been more knowledgeable on insomnia. Bias in our sample is also introduced through using vendor panel, as only those registered with the panel could be recruited. It is possible that views held among these providers are different than those who would have been recruited through a different method (e.g., random sampling). Providing participants with an incentive to participate may have also biased our sample. No formal statistical calculation of the sample size was performed and as this research did not collect demographic details on the sample the representativity of the PCPs population in each country is not guaranteed, which limit generalizability to other countries. Finally, it is important to note that our sample comprised PCPs who reported some experience with treating insomnia, and a sample with less experienced PCPs may not have rated the algorithm as favorably. However, the results contain valuable insights into an important aspect of improving quality of insomnia care; future research on this algorithm with a broader sample or using alternative recruitment strategies may mitigate the limitations stated.

## Conclusion

Chronic insomnia is a sleep disorder that affects nearly one in ten adults in Europe; however, diagnosis and management of insomnia through PCPs – the primary pathway for most patients – is suboptimal. There is a demonstrated need for support specifically tailored to PCPs to evaluate patients and determine the best pathway for the diagnosis of insomnia, in line with current guidelines. This research evaluating an algorithm to diagnose and manage insomnia showed that PCPs view such a tool favorably and felt it could positively impact their practice. Future efforts to evaluate this revised algorithm and its use in practice with patients in real-time would be beneficial for further refinement. There is also a need for PCPs to be more involved with efforts to improve chronic insomnia diagnosis and management through education, and for collaboration between researchers, specialist providers and PCPs to optimize care in the future.

### Electronic supplementary material

Below is the link to the electronic supplementary material.

Supplementary Material 1

Supplementary Material 2

Supplementary Material 3

Supplementary Material 4

Supplementary Material 5

## Data Availability

Data are provided within the manuscript or supplementary information files.

## References

[1] Morin C, Jarrin D (2022). Epidemiology of insomnia: prevalence, course, risk factors, and public health burden. Sleep Med Clin.

[2] Riemann D, Baglioni C, Bassetti C, Bjorvatn B, Dolenc Groselj L, Ellis JG (2017). European guideline for the diagnosis and treatment of insomnia. J Sleep Res.

[3] Li M, Zhang XW, Hou WS, Tang ZY (2014). Insomnia and risk of cardiovascular disease: a meta-analysis of cohort studies. Int J Cardiol.

[4] Li L, Wu C, Gan Y, Qu X, Lu Z (2016). Insomnia and the risk of depression: a meta-analysis of prospective cohort studies. BMC Psychiatry.

[5] Zhou F, Li S, Xu H (2022). Insomnia, sleep duration, and risk of anxiety: a two-sample mendelian randomization study. J Psychiatr Res.

[6] Shamim SA, Warriach ZI, Tariq MA, Rana KF, Malik BH, Insomnia. Risk Factor for Neurodegenerative Diseases. Cureus. 2019 Oct 26 [cited 2023 Jul 25]; https://www.cureus.com/articles/23253-insomnia-risk-factor-for-neurodegenerative-diseases. 10.7759/cureus.6004 PMC6876903 31807391

[7] Ellis J, Ferini-Strambi L, García-Borreguero D, Heidbreder A, O’Regan D, Parrino L (2023). Chronic insomnia disorder across Europe: Expert Opinion on challenges and opportunities to improve care. Healthcare.

[8] Cadet M, Tucker L, Allen D, Lawal E, Dickson D, Denis A (2019). Assessing for and managing chronic insomnia in primary care settings. The Nurse Practitioner.

[9] Ogeil RP, Chakraborty SP, Young AC, Lubman DI (2020). Clinician and patient barriers to the recognition of insomnia in family practice: a narrative summary of reported literature analysed using the theoretical domains framework. BMC Fam Pract.

[10] Riemann D, Espie CA, Altena E, Arnardottir ES, Baglioni C, Bassetti CLA (2023). The European Insomnia Guideline: an update on the diagnosis and treatment of insomnia 2023. J Sleep Res.

[11] Morin C (2017). Issues and challenges in implementing clinical practice guideline for the management of chronic insomnia. J Sleep Res.

[12] Rossman J (2019). Cognitive-behavioral therapy for Insomnia: an effective and underutilized treatment for Insomnia. Am J Lifestyle Med.

[13] Brandt J, Leong C (2017). Benzodiazepines and Z-Drugs: an updated review of major adverse outcomes reported on in Epidemiologic Research. Drugs R D.

[14] Soyka M, Wild I, Caulet B, Leontiou C, Lugoboni F, Hajak G (2023). Long-term use of benzodiazepines in chronic insomnia: a European perspective. Front Psychiatry.

[15] Palagini L, Manni R, Aguglia E, Amore M, Brugnoli R, Girardi P (2020). Expert opinions and Consensus recommendations for the evaluation and management of Insomnia in Clinical Practice: joint statements of five Italian Scientific societies. Front Psychiatry.

[16] Sateia MJ, Buysse DJ, Krystal AD, Neubauer DN, Heald JL (2017). Clinical practice Guideline for the Pharmacologic Treatment of Chronic Insomnia in adults: an American Academy of Sleep Medicine Clinical Practice Guideline. J Clin Sleep Med.

[17] Ellis JG, Gehrman P, Espie CA, Riemann D, Perlis ML (2012). Acute insomnia: current conceptualizations and future directions. Sleep Med Rev.

[18] Ellis J (2019). Cognitive behavioral therapy for insomnia and acute insomnia: considerations and controversies. Sleep Med Clin.

[19] Morin CM, Belleville G, Bélanger L, Ivers H (2011). The Insomnia Severity Index: psychometric indicators to detect insomnia cases and evaluate treatment response. Sleep.

[20] Passingham J, Baker R, Harding D, Cannon A. ESOMAR/GRBN Guideline for Reseachers and Clients Involved in Primary Data Collection. ESOMAR; https://esomar.org/uploads/attachments/cktim86vi054wsptru81egz40-guideline-on-primary-data-collection-final.pdf.

[21] Code of Conduct. Market Research Society (MRS). 2023 May [cited 2024 Mar 13]. https://www.mrs.org.uk/pdf/MRS-code-of-conduct-2023.pdf.

[22] 2023 Code of Conduct/AER. European Pharmaceutical Market Research Association; 2023 [cited 2024 Mar 13]. https://www.ephmra.org/code-conduct-aer.

[23] Legal and Ethical Guidelines. British Healthcare Business Intelligence Association. 2024 Feb [cited 2024 Mar 13]. https://www.bhbia.org.uk/guidelines-and-legislation/legal-and-ethical-guidelines.

[24] Jebb AT, Ng V, Tay L (2021). A review of Key Likert Scale Development advances: 1995–2019. Front Psychol.

[25] Liang G, Sloane JF, Donkin C, Newell BR (2022). Adapting to the algorithm: how accuracy comparisons promote the use of a decision aid. Cogn Res.

[26] Chima S, Reece JC, Milley K, Milton S, McIntosh JG, Emery JD (2019). Decision support tools to improve cancer diagnostic decision making in primary care: a systematic review. Br J Gen Pract.

[27] Harada T, Miyagami T, Kunitomo K, Shimizu T (2021). Clinical decision support systems for diagnosis in primary care: a scoping review. IJERPH.

[28] Ho S, Kalloniatis M, Ly A (2022). Clinical decision support in primary care for better diagnosis and management of retinal disease. Clin Experimental Optometry.

[29] Jernelöv S, Blom K (2023). Training of professionals in cognitive behavioural therapy for insomnia – a systematic review of peer-reviewed studies. J Sleep Res.

