# Supplementary material for: Assessment and management of chronic insomnia disorder: an algorithm for primary care physicians
Source: BMC Prim Care. 2024 Apr 26;25:138. doi: 10.1186/s12875-024-02381-w (PMC11055373; doi:10.1186/s12875-024-02381-w)
Supplement: Supplementary file 1 — Supplementary Material 1 [file 12875_2024_2381_MOESM1_ESM.docx]

**Appendix 1. Diagnosis and Management of Insomnia**

|  | Total  (n=106) | Germany  (n=22) | France  (n=21) | UK  (n=20) | Italy  (n=21) | Spain  (n=22) |
| --- | --- | --- | --- | --- | --- | --- |
| Regularly screen for chronic insomnia in patients | | | | | | |
| Yes | 45 (42%) | 7 (32%) | 8 (38%) | 8 (40%) | 7 (33%) | 15 (68%) |
| No | 55 (52%) | 14 (64%) | 12 (57%) | 12 (60%) | 12 (57%) | 5 (23%) |
| Don’t know | 6 (6%) | 1 (5%) | 1 (5%) | -- | 2 (10%) | 2 (9%) |
| Have enough time in consultations to address patients’ needs in relation to insomnia/trouble sleeping | | | | | | |
| Strongly agree | 8 (8%) | 4 (18%) | -- | 1 (5%) | 1 (5%) | 2 (9%) |
| Tend to agree | 29 (27%) | 5 (23%) | 3 (14%) | 5 (25%) | 11 (52%) | 5 (23%) |
| Neither agree nor disagree | 15 (14%) | 2 (9%) | 4 (19%) | 2 (10%) | 6 (29%) | 2 (9%) |
| Tend to disagree | 41 (39%) | 9 (41%) | 13 (62%) | 10 (50%) | 2 (10%) | 9 (41%) |
| Strongly disagree | 13 (12%) | 2 (9%) | 1 (5%) | 2 (10%) | 1 (5%) | 2 (9%) |
| Don’t know | -- | -- | -- | -- | -- | -- |
| Importance of treating insomnia compared to anxiety | | | | | | |
| Treating insomnia is much more important | 10 (9%) | 1 (5%) | 3 (14%) | 1 (5%) | 3 (14%) | 2 (9%) |
| Treating insomnia is slightly more important | 13 (12%) | 1 (5%) | 1 (5%) | 5 (25%) | 2 (10%) | 4 (18%) |
| Treating insomnia is equally important | 59 (56%) | 10 (45%) | 11 (52%) | 11 (55%) | 14 (67%) | 13 (59%) |
| Treating insomnia is slightly less important | 18 (17%) | 7 (32%) | 5 (24%) | 3 (15%) | -- | 3 (14%) |
| Treating insomnia is much less important | 2 (2%) | 2 (9%) | -- | -- | -- | -- |
| Don’t know | 4 (4%) | 1 (5%) | 1 (5%) | -- | 2 (10%) | -- |
| Importance of treating insomnia compared to depression | | | | | | |
| Treating insomnia is much more important | 4 (4%) | -- | 1 (5%) | 1 (5%) | 1 (5%) | 1 (5%) |
| Treating insomnia is slightly more important | 6 (6%) | 1 (5%) | 2 (10%) | 2 (10%) | -- | 1 (5%) |
| Treating insomnia is equally important | 61 (58%) | 11 (50%) | 11 (52%) | 13 (65%) | 11 (52%) | 15 (68%) |
| Treating insomnia is slightly less important | 20 (19%) | 5 (23%) | 4 (19%) | 4 (20%) | 5 (24%) | 2 (9%) |
| Treating insomnia is much less important | 12 (11%) | 4 (18%) | 2 (10%) | -- | 3 (14%) | 3 (14%) |
| Don’t know | 3 (3%) | 1 (5%) | 1 (5%) | -- | -- | -- |
| Feeling resourced to address patients’ needs in relation to insomnia/trouble sleeping | | | | | | |
| I am very well resourced | 20 (19%) | 7 (32%) | 3 (14%) | 1 (5%) | 6 (29%) | 3 (14%) |
| I am fairly well resourced | 64 (60%) | 13 (59%) | 10 (48%) | 12 (60%) | 12 (57%) | 17 (77%) |
| I am not very well resourced | 21 (20%) | 2 (9%) | 8 (38%) | 7 (35%) | 2 (10%) | 2 (9%) |
| I am not resourced at all | 1 (1%) | -- | -- | -- | 1 (5%) | -- |
| Don’t know | -- | -- | -- | -- | -- | -- |
| Perceived impact of a treatment algorithm flowchart on treatment of patients with insomnia/trouble sleeping |  |  |  |  |  |  |
| Very positive | 27 (25%) | 4 (18%) | 6 (29%) | 8 (40%) | 3 (14%) | 6 (27%) |
| Fairly positive | 68 (64%) | 16 (73%) | 9 (43%) | 11 (55%) | 16 (76%) | 16 (73%) |
| Neither positive nor negative | 10 (9%) | 2 (9%) | 5 (24%) | 1 (5%) | 2 (10%) | -- |
| Fairly negative | 1 (1%) | -- | 1 (5%) | -- | -- | -- |
| Very negative | -- | -- | -- | -- | -- | -- |
| Don’t know | -- | -- | -- | -- | -- | -- |
