# Supplementary material for: Assessment and management of chronic insomnia disorder: an algorithm for primary care physicians
Source: BMC Prim Care. 2024 Apr 26;25:138. doi: 10.1186/s12875-024-02381-w (PMC11055373; doi:10.1186/s12875-024-02381-w)
Supplement: Supplementary file 2 — Supplementary Material 2 [file 12875_2024_2381_MOESM2_ESM.docx]

**Appendix 2. Reactions to Algorithm**

|  | Total  (n=106) | Germany  (n=22) | France  (n=21) | UK  (n=20) | Italy  (n=21) | Spain  (n=22) |
| --- | --- | --- | --- | --- | --- | --- |
| The algorithm would help diagnose chronic insomnia patients |  |  |  |  |  |  |
| Strongly agree | 40 (38%) | 8 (36%) | 5 (24%) | 7 (35%) | 6 (29%) | 14 (64%) |
| Tend to agree | 52 (49%) | 12 (55%) | 10 (48%) | 10 (50%) | 12 (57%) | 8 (36%) |
| Neither agree nor disagree | 7 (7%) | -- | 3 (14%) | 2 (10%) | 2 (10%) | -- |
| Tend to disagree | 5 (5%) | 1 (5%) | 2 (10%) | 1 (5%) | 1(5%) | -- |
| Strongly disagree | 1 (1%_ | -- | 1 (5%) | -- | -- | -- |
| Don’t know | 1 (1%) | 1 (5%) | -- | -- | -- | -- |
| The algorithm would speed up diagnosis of chronic insomnia |  |  |  |  |  |  |
| Strongly agree | 32 (30%) | 6 (27%) | 5 (24%) | 7 (35%) | 5 (24%) | 9 (41%) |
| Tend to agree | 49 (46%) | 12 (55%) | 10 (48%) | 10 (50%) | 8 (38%) | 9 (41%) |
| Neither agree nor disagree | 11 (10%) | 3 (14%) | -- | 1 (5%) | 6 (29%) | 1 (5%) |
| Tend to disagree | 10 (9%) | -- | 5 (24%) | 1 (5%) | 1 (5%) | 3 (14%) |
| Strongly disagree | 3 (3%) | 1 (5%) | 1 (5%) | -- | 1 (5%) | -- |
| Don’t know | 1 (1%) | -- | -- | 1 (5%) | -- | -- |
| The algorithm would help exclude the possibility of insomnia due to restless legs syndrome or obstructive sleep apnoea | | | | | | |
| Strongly agree | 31 (29%) | 9 (41%) | 2 (10%) | 5 (25%) | 5 (24%) | 10 (45%) |
| Tend to agree | 52 (49%) | 10 (45%) | 13 (62%) | 10 (50%) | 10 (48%) | 9 (41%) |
| Neither agree nor disagree | 17 (16%) | 3 (14%) | 1 (5%) | 5 (25%) | 5 (24%) | 3 (14%) |
| Tend to disagree | 5 (5%) | -- | 4 (19%) | -- | 1 (5%) | -- |
| Strongly disagree | 1 (1%) | -- | 1 (5%) | -- | -- | -- |
| Don’t know | -- | -- | -- | -- | -- | -- |
| The algorithm would help make the right treatment decisions for chronic insomnia | | | | | | |
| Strongly agree | 40 (37%) | 7 (32%) | 7 (33%) | 6 (30%) | 4 (19%) | 16 (73%) |
| Tend to agree | 42 (40%) | 9 (41%) | 6 (29%) | 12 (60%) | 11 (52%) | 4 (18%) |
| Neither agree nor disagree | 17 (16%) | 4 (18%) | 3 (14%) | 2 (10%) | 6 (29%) | 2 (9%) |
| Tend to disagree | 6 (6%) | 2 (9%) | 4 (19%) | -- | -- | -- |
| Strongly disagree | -- | -- | -- | -- | -- | -- |
| Don’t know | 1 (1%) | -- | 1 (5%) | -- | -- | -- |
| The algorithm would help improve clinical practice overall in relation to chronic insomnia | | | | | | |
| Strongly agree | 42 (40%) | 8 (36%) | 6 (29%) | 6 (30%) | 6 (29%) | 16 (73%) |
| Tend to agree | 45 (42%) | 10 (45%) | 9 (43%) | 14 (70%) | 9 (43%) | 3 (14%) |
| Neither agree nor disagree | 9 (8%) | 2 (9%) | 1 (5%) | -- | 3 (14%) | 3 (14%) |
| Tend to disagree | 8 (8%) | 1 (5%) | 5 (24%) | -- | 2 (10%) | -- |
| Strongly disagree | 1 (1%) | -- | -- | -- | 1(5%) | -- |
| Don’t know | 1 (1%) | 1 (5%) | -- | -- | -- | -- |
| The algorithm matches guidelines for the treatment of insomnia/trouble sleeping | | | | | | |
| Strongly agree | 34 (32%) | 9 (41%) | 4 (19%) | 5 (25%) | 5 (24%) | 11 (50%) |
| Tend to agree | 50 (47%) | 11 (50%) | 9 (43%) | 8 (40%) | 12 (57%) | 10 (45%) |
| Neither agree nor disagree | 14 (13%) | 10 (45%) | 5 (24%) | 5 (25%) | 4 (19%) | -- |
| Tend to disagree | 5 (5%) | -- | 2 (10%) | 1 (5%) | -- | -- |
| Strongly disagree | -- | -- | -- | -- | -- | -- |
| Don’t know | 3 (3%) | -- | 1 (5%) | 5 (5%) | -- | 1 (5%) |
