# Supplementary material for: Assessment and management of chronic insomnia disorder: an algorithm for primary care physicians
Source: BMC Prim Care. 2024 Apr 26;25:138. doi: 10.1186/s12875-024-02381-w (PMC11055373; doi:10.1186/s12875-024-02381-w)
Supplement: Supplementary file 3 — Supplementary Material 3 [file 12875_2024_2381_MOESM3_ESM.docx]

**Appendix 3. Utility of Algorithm**

|  | Total  (n=106) | Germany  (n=22) | France  (n=21) | UK  (n=20) | Italy  (n=21) | Spain  (n=22) |
| --- | --- | --- | --- | --- | --- | --- |
| Usefulness– Page 1 |  |  |  |  |  |  |
| Very useful | 36 (34%) | 6 (27%) | 7 (33%) | 7 (35%) | 7 (33%) | 9 (41%) |
| Fairly useful | 62 (58%) | 13 (59%) | 12 (57%) | 13 (65%) | 13 (62%) | 11 (50%) |
| Not very useful | 8 (8%) | 3 (14%) | 2 (10%) | -- | 1 (5%) | 2 (9%) |
| Not useful at all | -- | -- | -- | -- | -- | -- |
| Don’t know | -- | -- | -- | -- | -- | -- |
| Usefulness– Page 2 |  |  |  |  |  |  |
| Very useful | 31 (29%) | 5 (23%) | 6 (29%) | 8 (40%) | 4 (19%) | 8 (36%) |
| Fairly useful | 59 (56%) | 11 (50%) | 10 (48%) | 11 (55%) | 14 (67%) | 15 (59%) |
| Not very useful | 15 (14%) | 6 (27%) | 4 (19%) | 1 (5%) | 3 (14%) | 1 (5%) |
| Not useful at all | 1 (1%) | -- | 1 (5%) | -- | -- | -- |
| Don’t know | -- | -- | -- | -- | -- | -- |
| Level of difficulty to use– Page 1 |  |  |  |  |  |  |
| Very easy | 17 (16%) | 2 (9%) | 5 (24%) | 3 (15%) | 3 (14%) | 4 (18%) |
| Fairly easy | 66 (62%) | 11 (50%) | 13 (62%) | 14 (70%) | 13 (62%) | 15 (68%) |
| Neither difficult nor easy | 19 (18%) | 8 (36%) | 2 (10%) | 2 (10%) | 4 (19%) | 3 (14%) |
| Fairly difficult | 3 (3%) | 1(5%) | -- | 1 (5%) | 1 (5%) | -- |
| Very difficult | 1 (1%) | -- | 1 (5%) | -- | -- | -- |
| Don’t know | -- | -- | -- | -- | -- | -- |
| Level of difficulty to use– Page 2 |  |  |  |  |  |  |
| Very easy | 14 (13%) | 2 (9%) | 3 (14%) | 2 (10%) | 2 (10%) | 5 (23%) |
| Fairly easy | 50 (47%) | 7 (32%) | 7 (33%) | 12 (60%) | 12 (57%) | 12 (55%) |
| Neither difficult nor easy | 21 (20%) | 5 (23%) | 6 (29%) | 2 (10%) | 5 (24%) | 3 (14%) |
| Fairly difficult | 17 (16%) | 8 (36%) | 2 (10%) | 4 (20%) | 1 (5%) | 2 (9%) |
| Very difficult | 4 (4%) | -- | 3 (14%) | -- | 1 (5%) | -- |
| Don’t know | -- | -- | -- | -- | -- | -- |
