# Supplementary material for: Assessment and management of chronic insomnia disorder: an algorithm for primary care physicians
Source: BMC Prim Care. 2024 Apr 26;25:138. doi: 10.1186/s12875-024-02381-w (PMC11055373; doi:10.1186/s12875-024-02381-w)
Supplement: Supplementary file 4 — Supplementary Material 4 [file 12875_2024_2381_MOESM4_ESM.docx]

**Appendix 4. Improvements to Algorithm**

|  | Total*  (n=35) | Germany  (n=6) | France  (n=7) | UK  (n=9) | Italy  (n=6) | Spain  (n=7) |
| --- | --- | --- | --- | --- | --- | --- |
| *Improvements to Algorithm - Page 1* |  |  |  |  |  |  |
| Content | 14 (40%) | 3 (50%) | 4 (57%) | 1 (11%) | 2 (33%) | 4 (57%) |
| Convenience | 10 (29%) | 3 (50%) | 2 (29%) | 1 (11%) | 2 (33%) | 2 (29%) |
| Text/wording | 4 (11%) | 2 (33%) | -- | -- | 1 (17%) | 1 (14%) |
| Information for treatment | 4 (11%) | -- | 1 (14%) | -- | -- | 3 (43%) |
| Layout | 2 (6%) | 2 (33%) | -- | -- | -- | -- |
| Usage in clinical practice | 8 (23%) | 3 (50%) | 2 (29%) | -- | -- | 3 (43%) |
| Other usage in clinical practice | 7 (20%) | 2 (33%) | 2 (29%) | -- | -- | 3 (43%) |
| Patient types | 3 (9%) | 2 (33%) | 1 (14%) | -- | -- | -- |
| Appeal | 5 (14%) | 2 (33%) | 1 (14%) | 1 (11%) | -- | 1 (14%) |
| Visuals | 3 (9%) | -- | 2 (29%) | -- | 1 (17%) | -- |
| Patient compliance/preference | 3 (9%) | -- | 1 (14%) | -- | 1 (17%) | 1 (14%) |
| Miscellaneous – digitize algorithm | 2 (6%) | 1 (17%) | -- | -- | 1 (17%) | -- |
| *Improvements to Algorithm - Page 2* |  |  |  |  |  |  |
| Content | 21 (60%) | 3 (50%) | 3 (43%) | 6 (67%) | 5 (83%) | 4 (57%) |
| Convenience | 15 (43%) | 1 (17%) | 2 (29%) | 4 (44%) | 5 (83%) | 3 (43%) |
| Text/wording | 1 (3%) | -- | -- | 1 (11%) | -- | -- |
| Information for treatment | 3 (9%) | -- | -- | 2 (22%) | -- | 1 (14%) |
| Layout | 10 (29%) | 2 (33%) | 1 (14%) | 5 (56%) | 1 (17%) | 1 (14%) |
| Usage in clinical practice | 7 (20%) | 1 (17%) | -- | 2 (22%) | 2 (33%) | 1 (14%) |
| Other usage in clinical practice | 6 (17%) | 1 (17%) | -- | 2 (22%) | 2 (33%) | 1 (14% |
| Patient types | 3 (9%) | 1 (17%) | -- | 1 (11%) | -- | 1 (14%) |

**Sub-set of sample who were preassigned to respond to open-ended questions about tool improvements*
