# Supplementary material for: Assessment and management of chronic insomnia disorder: an algorithm for primary care physicians
Source: BMC Prim Care. 2024 Apr 26;25:138. doi: 10.1186/s12875-024-02381-w (PMC11055373; doi:10.1186/s12875-024-02381-w)
Supplement: Supplementary file 5 — Supplementary Material 5 [file 12875_2024_2381_MOESM5_ESM.docx]

**Appendix 5. Chronic Insomnia HCP Research Algorithm Screener and Questionnaire**

**Screening Criteria:**

**[FOR REFERENCE ONLY]**

| SSpecialty. | Must be a PCP |
| --- | --- |
| SYears. | Must have been in current specialty/role between 3 and 35 years |
| SClinicalPractice. | Must spend at least 70% of time seeing patients |
| STreat2. | Must see at least 20 adult insomnia patients in a typical month |
| Streat3. | Must treat at least 10 insomnia patients a month with prescription therapy |

# **Screener**

SCountry. In which country do you live?

(Please select one answer only)

- France
- Germany
- Spain
- Italy
- UK
- None of these

[PN: SINGLE CODE. CLOSE IF ‘NONE OF THESE’ SELECTED. PRE-PUNCH FROM PANEL, DO NOT SHOW TO RESPONDENTS]

SIntro. This survey is being conducted by Ipsos, an independent market research agency based in the UK, on behalf of a pharmaceutical company. We are conducting research on insomnia treatment and would like to ask you some questions on this topic. The survey will take approximately **[PN: QUANT** 10**]** **[PN: QUAL** 20**]** minutes of your time. You will receive an incentive for your participation.

With your consent, your information will only be collected and used for market research and analysis. Any information you give will be treated in the strictest confidence and results will only be reported back on an aggregated basis. Answers may be used in external publications. We may need to send some of your personal data outside of the UK or your country of residence. We will make sure that it is kept secure at all times.

As a member of the Market Research Society (MRS), Ipsos is bound by the MRS Code of Conduct and all applicable laws protecting your personal data and responses. The study is conducted in compliance with MRS/ ESOMAR/ EphMRA [PN: UK ONLY - / British Healthcare Business Intelligence Association] guidelines and codes of conduct. You have the right to withdraw from the interview at any time. For more information about your rights and how data will be used, please see our privacy notice, it is available here [PN: SCRIPTERS TO INSERT PRIVACY POLICY LINK].

Are you happy to participate in this research as set out above and in the privacy notice?

(Please select one answer only)

- Yes, I wish to continue
- No, I do not wish to continue

**[PN: SINGLE CODE. CLOSE IF ‘NO’ SELECTED. IF CLOSED]**

**[PN: SHOW ON NEXT SCREEN, ASK IF QUAL QUOTA HASN’T BEEN MET YET]** During this survey you will have the option to answer certain questions by capturing a voice recording. This enables us to provide in-depth, meaningful analysis to our client.

Are you happy to provide an audio response?

(Please select one answer only)

- I am happy to provide an audio response **[PN: ASSIGN TO QUAL QUOTA]**
- I’d like to skip that task

SData. As you are aware the Ministère des Affaires sociales et de la Santé is requesting more transparency from pharmaceutical companies on payments made to doctors. This means market research companies are now obliged to provide details of the name and honorarium amount paid to physicians taking part in market research. Pursuant to Article L.1453-1 of the Code of Public Health and Regulatory Decree No. 2013-414 of 21 May 2013 on the transparency of benefits provided by the companies producing or marketing products health and cosmetic purposes for humans, certain information relating to this agreement will be published on a dedicated French Health minister website. Pursuant to Decree No. 2013-414 of application you do not have the right to object to the publication of such personal data. Are you happy to proceed on this basis?

(Please select one answer only)

- Yes
- No

[PN: SHOW ONLY IN FR. SINGLE CODE. CLOSE IF ‘NO’ SELECTED]

SSpecialty. Which of the following best describes your primary specialty?

(Please select one answer only)

- Psychiatrist [PN: TERMINATE]
- Neurologist [PN: TERMINATE]
- **ITALY/ UK/ GERMANY/ FRANCE:** General practitioner / **SPAIN ONLY:** Family Medicine
- Other (please specify) [PN: TERMINATE]

[PN: ASK ALL]

SYears. Approximately how many **years** have you been qualified in your current specialty?

(Please type in your answer)

_______years qualified

[PN: ASK ALL, OPEN NUMERIC RANGE 0 TO 70]

[PN: TERMINATE IF LESS THAN 3 OR MORE THAN 35]

SClinicalPractice. In a typical week, what percentage of your professional time do you spend in clinical practice treating patients (as opposed to time spent in clinical research, teaching or administrative duties)?

(Please type in your answer)

________% of professional time seeing patients

[PN: ASK ALL, OPEN NUMERIC % - RANGE 0% TO 100%]

[PN: TERMINATE IF LESS THAN 70%]

**STreat2.** In a typical month, approximately how many adult patients with insomnia do you personally manage for their insomnia, if any?

Please refer to your chronic insomnia patients only, not to those suffering it due to their underlying mental health conditions

(Please type in your answer)

________adult patients with chronic insomnia

[PN: ASK ALL, OPEN NUMERIC]

[TERMINATE IF LESS THAN 20]

Streat3. Of the [INSERT FROM STreat2] adult patients you treat with insomnia in a typical month, for how many do **you personally prescribe** treatment for their insomnia?

(Please type in your answer)

____ patients I personally prescribe prescription therapy to in a typical month

[PN: ASK ALL, TERMINATE IF LESS THAN 10]

# **QUESTIONNAIRE**

# **Section A: Pre-exposure Questions**

Thank you. We can confirm that you are eligible to take part in the rest of this survey. We would like to start by asking you questions regarding consultations with your patients in relation to their insomnia / trouble sleeping.

A1. To what extent, if at all, do you feel patients effectively communicate their insomnia / trouble sleeping symptoms?

(Please select one answer only)

- Not at all
- Hardly at all
- To some extent
- To a great extent
- Don’t know

**[PN: ASK ALL, SINGLE CODE]**

A2. In cases of patients suffering with anxiety and patients suffering with depression in addition to insomnia / trouble sleeping, which condition is more important to treat?

(Please select one answer per column)

**[PN: COLUMNS]**

- Anxiety
- Depression

**[PN: ROWS]**

- Treating insomnia / trouble sleeping is much more important than…
- Treating insomnia / trouble sleeping is slightly more important than…
- Treating insomnia / trouble sleeping is equally as important as…
- Treating insomnia / trouble sleeping is slightly less important than…
- Treating insomnia / trouble sleeping is much less important than…
- Don’t know

**[PN: ASK ALL, SINGLE CODE]**

A3. To what extent do you agree or disagree that patients understand your treatment advice in relation to insomnia / trouble sleeping?

(Please select one answer only)

- Strongly disagree
- Tend to disagree
- Neither agree not disagree
- Tend to agree
- Strongly agree
- Don’t know

**[PN: ASK ALL, SINGLE CODE]**

A4. To what extent do you agree or disagree that patients adhere to your advice in relation to insomnia / trouble sleeping?

(Please select one answer only)

- Strongly disagree
- Tend to disagree
- Neither agree not disagree
- Tend to agree
- Strongly agree
- Don’t know

**[PN: ASK ALL, SINGLE CODE]**

A5. To what extent, if at all, do you feel resourced to address patients’ needs in relation to insomnia / trouble sleeping?

(Please select one answer only)

- I am not resourced at all
- I am not very well resourced
- I am fairly well resourced
- I am very well resourced
- Don’t know

**[PN: ASK ALL, SINGLE CODE]**

A6A. Do you regularly screen for chronic insomnia in your patients?

(Please select one answer only)

- Yes – I regularly screen for chronic insomnia in my patients
- No – I do not regularly screen for chronic insomnia in my patients
- Don’t know

**[PN: ASK ALL, SINGLE CODE]**

A6B. Do you agree or disagree with the following statement: “I have enough time in consultations to address my patients’ needs in relation to insomnia/ trouble sleeping”?

(Please select one answer only)

- Strongly disagree
- Tend to disagree
- Neither agree nor disagree
- Tend to agree
- Strongly agree
- Don’t know

**[PN: ASK ALL, SINGLE CODE]**

A7. To what extent, if at all, are patients involved in decisions around their insomnia / trouble sleeping treatment?

(Please select one answer only)

- Not at all
- Hardly at all
- To some extent
- To a great extent
- Don’t know

**[PN: ASK ALL, SINGLE CODE]**

A8A. Please now imagine a tool that patients with chronic insomnia could use to record their symptoms & expectations for their consultations with healthcare professionals

Do you believe that such a tool would have a positive or negative impact on patients’ ability to communicate with you in relation to their insomnia / trouble sleeping?

(Please select one answer only)

- Very negative
- Fairly negative
- Neither positive nor negative
- Fairly positive
- Very positive
- Don’t know

**[PN: ASK ALL, SINGLE CODE]**

A8B. How often, if at all, would you ask your patients to use this tool before consultations?

(Please select one answer only)

- Never
- Hardly
- Frequently
- Always
- Don’t know

**[PN: ASK ALL, SINGLE CODE]**

# **Section B: Patient Tool**

We would now like to show you a tool for your patients that has been designed to better identify symptoms and improve the quality of conversations they have with you.

**[PN: SHOW PATIENT TOOL – FORCE RESPONDENTS TOOL FOR AT LEAST 20 SECONDS]**

**[PN: SHOW THIS LINE OF TEXT ON EACH PAGE OF THIS SECTION – INSERT HYPERLINK TO THE TOOL]** Please click here to view the patient tool again.

B1. To what extent do you agree or disagree that this tool would:

(Please select one answer per row)

**[PN: COLUMNS]**

- Strongly disagree
- Tend to disagree
- Neither agree nor disagree
- Tend to agree
- Strongly agree
- Don’t know

**[PN: ROWS]**

- Help your patients communicate with you in relation to their insomnia / trouble sleeping?
- Help your patients monitor their insomnia / trouble sleeping in between visits?
- Help patients use consultation time with you optimally in relation to insomnia / trouble sleeping
- Help you have efficient conversations with your insomnia / trouble sleeping patients

**[PN: ASK ALL, SINGLE CODE PER ROW, RANDOMISE]**

B2. Do you think the tool would be easy or difficult for insomnia / trouble sleeping patients to use?

(Please select one answer only)

- Very difficult to use
- Fairly difficult to use
- Neither difficult nor easy to use
- Fairly easy to use
- Very easy to use
- Don’t know

**[PN: ASK ALL, SINGLE CODE]**

B3. Do you feel there is anything missing from the tool?

(Please select one answer only)

- Yes, please specify **[PN: TEXT BOX]**
- No
- Don’t know

**[PN: ASK ALL, SINGLE CODE]**

B7. You previously mentioned you don’t think this tool would help you to have more efficient conversations with your insomnia patients/ patients who have trouble sleeping. Why is that?

(Please select all that apply)

- The list of symptoms is not comprehensive enough
- It is unclear how it works
- I do not like the design
- There is too much information
- Other, please specify **[PN: FIX]**
- I don’t know **[PN: FIX, EXCLUSIVE]**

**[PN: ASK THOSE SELECTING ‘STRONGLY/TEND TO DISAGREE’ FOR ‘HELP YOU HAVE MORE EFFICIENT CONVERSATIONS WITH YOUR INSOMNIA/TROUBLE SLEEPING PATIENTS’ AT B1, MULTICODE, RANDOMISE]**

Section C: HCP Tool

A9. Now imagine a treatment algorithm flowchart for HCPs such as yourself to use to guide the management of insomnia.

Do you believe that such a tool would have a positive or negative impact on your treatment of patients with insomnia / trouble sleeping?

(Please select one answer only)

- Very negative
- Fairly negative
- Neither positive nor negative
- Fairly positive
- Very positive
- Don’t know

**[PN: ASK ALL, SINGLE CODE]**

We would now like to show you an algorithm to assist you in diagnosing and treating patients with insomnia / trouble sleeping.

**[PN: SHOW HCP TOOL – FORCE RESPONDENTS TO VIEW EACH PAGE OF THE TOOL FOR AT LEAST 5 SECONDS]**

**[PN: SHOW THIS LINE OF TEXT ON EACH PAGE OF C1 AND C2 – INSERT HYPERLINK TO THE TOOL]** Please click here to view the algorithm again.

C1. Please use the link below to record a short audio explaining how you would improve the tool **overall.**

Please give any suggestions that come to mind, this could include but is not limited to layout, content and wording.

Please click on the button below and speak into your microphone to record your answer.

**[PN: ASK TO QUAL ONLY, INSERT BUTTON TO RECORD AUDIO]**

C2. To what extent do you agree or disagree that this tool:

(Please select one answer per row)

**[PN: COLUMNS]**

- Strongly disagree
- Tend to disagree
- Neither agree nor disagree
- Tend to agree
- Strongly agree
- Don’t know

**[PN: ROWS]**

- Would help you diagnose chronic insomnia patients
- Would speed up the diagnosis of chronic insomnia
- Would help you exclude the possibility of insomnia due to restless legs syndrome (RLS) or obstructive sleep apnoea (OSA)?
- Would help you make the right treatment decisions for chronic insomnia?
- Would help improve your clinical practice overall in relation to chronic insomnia?
- Would be easy to use
- Matches guidelines for the treatment of insomnia / trouble sleeping?

**[PN: ASK ALL, SINGLE CODE PER ROW, RANDOMISE]**

**[PN: SHOW A PAGE OF THE HCP TOOL AND REPEAT C10-C12 FOR EACH PAGE OF THE TOOL]**

**[PN: SHOW THIS LINE OF TEXT ON EACH PAGE OF C10 TO C12 – INSERT HYPERLINK TO THE TOOL]** Please click here to view the section of the algorithm again.

**C10.** How useful, if at all, do you find this section of the tool?

(Please select one answer only)

- Not useful at all
- Not very useful
- Fairly useful
- Very useful
- Don’t know

**[PN: ASK ALL, SINGLE CODE]**

**C11.** To what extent do you find this section of the tool easy or difficult to use?

(Please select one answer only)

- Very difficult
- Fairly difficult
- Neither difficult nor easy
- Fairly easy
- Very easy
- Don’t know

**[PN: ASK ALL, SINGLE CODE]**

C12. Please use the link below to record a short audio explaining how you would improve this section of the tool.

Please give any suggestions that come to mind, this could include but is not limited to layout, content and wording.

Please click on the button below and speak into your microphone to record your answer.

**[PN: ASK TO QUAL ONLY, INSERT BUTTON TO RECORD AUDIO]**

# **Section D: Pharmacovigilance Re-contact**

Thank you for your time. We have 1-2 simple questions to finish.

**D1.** The sponsoring company of this research has an obligation to continuously monitor the safety of their products. Although rare, their Product Safety Department may want to further investigate any issues you may have raised in this survey. If we identify any adverse event/product complaint mentioned by you during this research, are you willing to be contacted by us to provide more information regarding the adverse event/product complaint?

(Please select one answer only)

- Yes, I am willing to be contacted to provide more information regarding the adverse event/product complaint
- No, I am not willing to be contacted to provide more information regarding the adverse event/product complaint

**[PN: SINGLE CODE. SHOW TO QUAL RESPONDENTS ONLY]**

**D2.** Would you be willing to have your personal data passed on to the sponsoring company for the purpose of obtaining more information regarding any adverse event / product complaints mentioned in this research? Please note that if you were to consent to having your personal data passed on to the sponsoring company, Idorsia Pharmaceuticals, such personal data will be controlled and processed by their Product Safety Department. Their privacy notice that can be reviewed at this link https://www.idorsia.com/system/legal-statements/privacy-policy.

(Please select one answer only)

- Yes, I consent to my personal data being passed on to Idorsia Pharmaceuticals.
- No, I do not consent to my personal data being passed on to Idorsia Pharmaceutica.

**[PN: SINGLE CODE. USE FOR OFF-LINE RE-CONTACT OF RESPONDENTS TO ASK FOR CONSENT TO PASS ON PERSONAL DATA TO CLIENT AS REQUIRED FOR QUAL RESPONDENTS ONLY. DO NOT SHOW IN GERMANY]**

**F3.** As mentioned at the beginning of the survey we can now reveal that this research is sponsored by Idorsia Pharmaceuticals. Please view here https://www.idorsia.com/system/legal-statements/privacy-policy if you would like to find out more information about how the sponsoring company processes personal data.

**[PN: SHOW IN GERMANY ONLY]**
